# Spatial Distributions of Active Pico- and Nano-Haptophytes (Eukaryota, Hacrobia) in the Tropical and Subtropical Western Pacific Ocean

**DOI:** 10.3390/microorganisms14040941

**Published:** 2026-04-21

**Authors:** Wenlu Li, Yuyu Liao, Nianzhi Jiao, Dapeng Xu

**Affiliations:** 1 State Key Laboratory of Marine Environmental Science, Institute of Marine Microbes and Ecospheres, College of Ocean and Earth Sciences, Xiamen University, Xiamen 361102, China; lwl5493658@163.com (W.L.); 22320171150829@xmu.edu.cn (Y.L.); jiao@xmu.edu.cn (N.J.); 2 Fujian Key Laboratory of Marine Carbon Sequestration, Xiamen University, Xiamen 361102, China

**Keywords:** community assembly, co-occurrence network, high-throughput sequencing, intermediate taxa, SSU rRNA

## Abstract

Haptophytes are ubiquitous single-celled eukaryotic plankton in coastal and open oceans that play a key role in marine biogeochemical cycling. Understanding the size structure and community composition of active haptophytes is crucial for elucidating their diversity and ecological functions. This study investigated the diversity and community structure of pico- (0.2–3 μm) and nano-sized (3–20 μm) haptophytes in the surface waters of the western Pacific Ocean using high-throughput sequencing targeting the hypervariable V4 region of the 18S rRNA. The pico-sized community exhibited significantly higher diversity than the nano-sized community. Community composition varied significantly between size fractions, driven primarily by the genera *Chrysochromulina* and *Syracosphaera*. Furthermore, the nano-sized community was more strongly influenced by environmental variables than the pico-sized community, although neither size fraction displayed a clear coastal-to-open-ocean distribution pattern. Null and neutral community model analyses indicated that both size-fractionated communities were primarily regulated by stochastic processes, while deterministic processes exerted a greater influence on the nano-sized community. Co-occurrence network analysis revealed stronger interconnections and a higher number of keystone species within the nano-sized community. In both networks, intermediate taxa (relative abundances of 0.01% to 0.1%) exhibited the highest diversity and abundance among keystone species, highlighting their pivotal role in shaping the network structure and stability. Additionally, phylogenetic analyses revealed that while the majority of ZOTUs clustered with known taxa, multiple deep-branching, uncultured lineages were identified across both size fractions, indicating substantial uncharacterized genetic diversity. This study underscores the variability and hidden diversity of size-fractionated haptophyte community structures in oligotrophic open oceans, providing valuable insights into their functional significance in global biogeochemical cycles.

## 1. Introduction

Haptophyta (Eukaryota, Hacrobia) is a division of unicellular eukaryotic algae characterized by four-membrane plastids that are derived from a red alga, which itself originated from the primary endosymbiosis of a cyanobacterium by a phagotrophic eukaryote [1]. Haptophytes play a pivotal role in marine microbial food webs and global biogeochemical cycles [2,3,4,5]. Primarily photoautotrophic, they contribute substantially to marine primary production. For example, small prymnesiophytes have been shown to contribute significantly to CO_2_ fixation in the subtropical and tropical northeast Atlantic Ocean [6]. Furthermore, mixotrophy, which integrates photoautotrophy and phagotrophy, enhances their survival in oligotrophic or low-light conditions and improves carbon transfer efficiency relative to strict autotrophic or heterotrophic counterparts [7,8,9]. Calcifying haptophytes further influence the carbon cycle by sequestering inorganic carbon via calcification, thereby promoting vertical carbon flux [10,11,12]. Previous studies have also demonstrated that some haptophytes host the symbiotic nitrogen-fixing cyanobacteria UCYN-A, enhancing overall nitrogen fixation efficiency [13,14,15,16]. A recent study confirmed that UCYN-A has evolved beyond endosymbiosis to function as an early-stage N_2_-fixing organelle, or “nitroplast” [17]. Additionally, haptophyte blooms facilitate sulfur cycling through the production of dimethylsulfoniopropionate (DMSP) [18]. Together, these distinctive ecological functions make haptophytes essential contributors to marine biogeochemical cycles.

Haptophytes exhibit a widespread distribution across global oceans, as evidenced by diverse methodologies. High performance liquid chromatography (HPLC) analyses of phytoplankton specific accessory pigments have highlighted the prevalence of these eukaryotic phytoplankton. Specifically, 19-hexanoyloxyfucoxanthin (19-Hex), an accessory photosynthetic pigment found exclusively in chloroplasts of haptophyte origin, predominates in the photic zone of open ocean waters [19,20,21] and can account for a major portion of the total Chl *a* biomass [22,23]. For instance, in the western Pacific Ocean (wPO), diagnostic pigment of haptophytes constituted 57–74% of the total eukaryotic algae pigments in surface waters [23]. Haptophytes are a crucial component of the phytoplankton community in the South China Sea [24,25]. Globally, it is estimated that haptophytes contribute approximately twice as much to the oceanic Chl *a* standing stock in the photic zone as either cyanobacteria or diatoms [21]. Consistent with the pigment analysis, the application of FISH-TSA (fluorescence in situ hybridization coupled with tyramide signal amplification) using haptophyte-specific probes has further documented their abundance and cell size composition in coastal and open ocean waters. These findings underscore their broad distribution and occasional dominance over eukaryotic phytoplankton across diverse marine environments [20,26,27,28]. The extensive application of high-throughput sequencing techniques targeting marker genes has enhanced our understanding of the temporal and spatial distribution, community composition, and assembly processes of haptophytes across diverse marine environments [29,30,31,32,33,34]. Recent global ocean estimates rank haptophytes as the second most abundant eukaryotic phytoplankton, following diatoms [35]. Unlike large heterotrophs (e.g., copepods and tunicates) that exhibit a basin-based community structure, haptophytes display a latitude-based structure at a smaller spatial scale [36]. At the basin scale, the assembly processes of haptophytes are primarily driven by heterogeneous selection, which is largely controlled by nutrient availability [37]. In terms of large-scale distribution, Prymnesiales (especially members of *Chrysochromulina*) dominated most *Tara* Oceans stations, with the exception of the Southern Ocean, where sequences affiliated with Phaeocystales were most prevalent. Other lineages, including CSZ, Isochrysidales, and Pavlovophyceae, exhibited generally low global abundances, despite occasional regional peaks [5]. Regarding temporal dynamics, a two-year study at a station in the outer Oslofjorden, Norway, revealed that haptophyte species richness peaked in autumn and reached its lowest point in spring. While certain taxa, such as *Chrysochromulina simplex*, *Emiliania huxleyi* and *Phaeocystis cordata*, were detected year-round, most calcifying coccolithophores appeared exclusively from summer to early winter [30].

Traditionally, the identification of haptophytes has relied on microscopic observations of isolated, cultured samples [38]. Currently, the division Haptophyta comprises 7 orders, 21 families, and 83 genera [39]. Approximately 300 to 400 species have been morphologically described, predominantly falling within the nano-sized (2/3–20 μm) fraction [39,40]. However, environmental sequencing studies have unveiled considerable, previously overlooked diversity within this group [31,39]. Several environmental clades consisting entirely of uncultured ribotypes have been reported, such as Clades B3–B5, Clades D–F, and Clades HAP1 to HAP5 [40]. Phytoplankton cell size is widely recognized as a key determinant of spatial distribution, significantly impacting aquatic ecosystem interactions and shaping diverse ecological functions [41]. Consequently, the composition of size fractionated haptophyte communities warrants in-depth investigation. Despite recent advances in characterizing overall haptophyte diversity, studies on the ecological distribution of these size-fractionated communities remain limited, particularly regarding their environmental drivers, assembly processes, and co-occurrence networks. A deeper understanding of these dynamics is vital for elucidating their niche partitioning, biogeochemical impacts, and responses to environmental fluctuations.

This study investigated active haptophyte communities in the surface waters of the tropical/subtropical wPO using size-fractionated filtration and high-throughput sequencing of the hypervariable V4 regions of the 18S rRNA gene. Specifically, our study aimed to determine: (1) how community structure differs between pico- and nano-haptophyte communities, (2) which environmental variables shape these two size fractions, (3) what the underlying community assembly processes are for pico- and nano-haptophyte communities, and (4) how their co-occurrence networks differ.

## 2. Materials and Methods

### 2.1. Sample Collection and Measurement on Environmental Parameters

**Figure 1:**
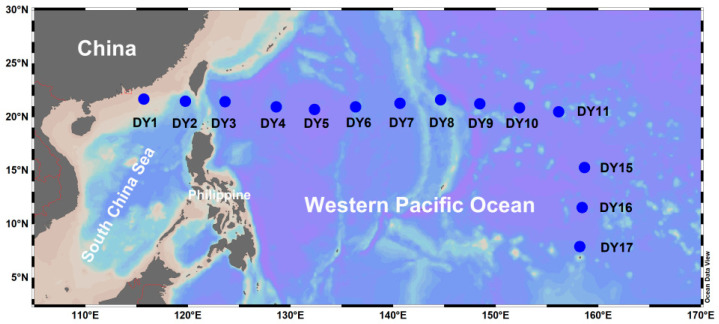
Map of the 14 sampling stations in the tropical and subtropical western Pacific Ocean.

Samples were collected from 14 stations in the subtropical/tropical wPO on board the *R/V* Xiang Yang Hong (leg DY40B) from 5 August to 16 October 2016 (Figure 1). Protocols for sample collection and the measurement of environmental parameters, including seawater temperature (T), salinity (S), nutrients (nitrite+ nitrate, NO_2_ + NO_3_; phosphate, PO_4_; and silicate, Si), phytoplankton pigment composition, and the abundances of picoplankton (heterotrophic bacteria, *Synechococcus*, *Procholorococcus*, and pigmented picoeukaryotes), nanoflagellates (heterotrophic nanoflagellates and pigmented nanoflagellates), and ciliate, are detailed in Huang et al. [42].

For environmental sequencing, surface seawater was collected using a plastic barrel and pre-filtered through a 200 μm mesh sieve to remove large plankton. Approximately 5 L of this prefiltered seawater was then sequentially filtered through polycarbonate filter membranes (Millipore, Darmstadt, Germany) with pore sizes of 20 μm, 3 μm, and 0.4 μm to separate the pico- (0.4–3 μm), nano- (3–20 μm), and micro (20–200 μm)-fractions, respectively. Filtration was completed within 30 min of sample retrieval. The filter membranes were immediately flash-frozen in liquid nitrogen and stored at −80 °C until RNA extraction. For this study, only the pico- and nano-sized fractions were analyzed.

### 2.2. RNA Extraction, PCR Amplification, and High-Throughput Sequencing

Environmental RNA was extracted using the RNeasy Mini Kit (Qiagen, Germantown, MD, USA) following the manufacturer’s instructions. Carryover genomic DNA was removed via DNase treatment (Promega, Madison, WI, USA), and the purified RNA was subsequently reverse transcribed into cDNA using the QuantiTect^®^ Reverse Transcription Kit (Qiagen, Germantown, MD, USA). The V4 region (ca. 380 bp) of the 18S rRNA gene was amplified using the Haptophyta-specific primers 528Flong and PRYM01 + 7, with PCR conditions following those described by Egge et al. [43]. To obtain sufficient amplicons for sequencing, PCR was run in four separate reactions for each sample. The resulting PCR amplicons were excised from gels and purified using the Wizard^®^ SV Gel and PCR Clean-Up Kit (Promega, Shanghai, China). Paired-end sequencing (2 × 250 bp) was performed on an Illumina MiSeq platform at Majorbio (Shanghai, China). Sequences generated in this study have been deposited in the NCBI Sequence Read Archive (SRA) database under BioProject accession number PRJNA1243746.

### 2.3. Data Processing and Statistical Analyses

Raw reads were quality-filtered, demultiplexed, and assembled using Trimmomatic [44] and Flash [45] according to Li et al. [46]. Mothur (v.1.21.3) was used to retain reads between 300 and 500 bp in length [47]. Quality-filtered reads were dereplicated using Usearch 11 [48]. Reads were then denoised and clustered into biological zero-radius operational taxonomic units (ZOTUs), which is defined as sequence clusters with 100% similarity, using UNOISE3 (v.11) [49]. Taxonomic annotation of the resulting ZOTUs was performed with SINTAX [50] against the Protist Ribosomal Reference (PR2) database (version 4.11.1), which integrates a curated haptophyte reference database [51]. Non-haptophyte ZOTUs and singletons were removed prior to downstream analysis. To ensure reliability and accuracy of alpha- and beta-diversity comparisons among samples, data were randomly resampled by the minimum number of reads across all samples. Alpha diversity indices, including ZOTU richness, the Shannon index, and Faith’s phylogenetic diversity (PD), were calculated using QIIME (v.1.9.0) [52]. Differences between sample groups were evaluated via analysis of similarity (ANOSIM) using the “vegan” package in R. Beta-diversity was visualized using principal coordinated analysis (PCoA) ordination based on a Bray–Curtis dissimilarities matrix and unweighted UniFrac distances using the “vegan” package in R (v.4.2.1).

ZOTUs were classified into three categories depending on their relative sequence abundance across the regional community (i.e., the composite of all sites). Abundant taxa (AT) were defined as ZOTUs comprising 0.1% or more of the total sequences. ZOTUs with relative abundances at or below 0.01% were categorized as rare taxa (RT). The remaining ZOTUs were classified as intermediate taxa (IT) [53,54,55].

Similarity percentage analysis (SIMPER) was performed in PRIMER6 (v.6.0) [56] to identify the ZOTUs contributing most to community dissimilarity between the two size fractions. Relationships between haptophyte communities and environmental factors were assessed using Mantel tests. Variance partitioning analysis (VPA) was used to determine the relative importance of environmental, biological, and spatial variables in shaping the size-fractionated communities. Both the Mantel tests and VPA were conducted using the “vegan” package in R.

### 2.4. Community Assembly Process

The null model (NM) was used to determine the potential contribution of different ecological processes [57]. Pairwise phylogenetic turnover was calculated using the weighted *β*-mean nearest taxon distance (*β*MNTD), which evaluates the phylogenetic distance between ZOTUs. This procedure was repeated 999 times to generate a distribution of null values. To measure the significance of the difference between *β*MNTD and its expected value, the *β*-nearest taxon index (*β*NTI) was calculated as the number of standard deviations of the observed *β*MNTD deviated from the mean of the null distribution in units of SDs. The *β*NTI, combined with a Bray–Curtis-based Raup–Crick (RC_Bray_) was used to quantify the relative influence of major ecological processes governing the haptophyte communities [58]. An |*β*NTI| > 2 indicates deterministic processes (i.e., the non-random fraction), including homogeneous (*β*NTI < −2) and heterogeneous selection (*β*NTI > 2), while |*β*NTI| < 2 indicates stochasticity (i.e., the random fraction), including probabilistic dispersal (|RC_Bray_| > 0.95) and undominated processes (|RC_Bray_| < 0.95) [59].

The neutral community model (NCM) was used to determine the proportion of stochastic processes governing community assembly for the pico- and nano-sized haptophyte communities, respectively. The parameter *R*^2^ represents the overall fit to the neutral model, and *m* indicates the immigration rate [60]. The 95% confidence intervals around all fitting statistics were calculated via bootstrapping with 1000 replicates.

### 2.5. Co-Occurrence Network Analysis

To reduce dataset complexity, only ZOTUs including ≥10 reads and detected in ≥30% of samples in the pico- and nano-dataset, respectively, were selected for network construction. All possible pairwise Spearman’s rank correlations (*r*) between these ZOTUs were calculated in R. Only robust (*r* > 0.6 or *r* < −0.6) and statistically significant (*p* < 0.05) correlations were included in network analyses. In the constructed networks, each node represents a single ZOTU, and each edge corresponds to a strong, significant correlation between nodes. Network visualization, modular analysis, and the calculation of network topological parameters, including average degree, network diameter, network density, modularity, average clustering coefficient, and average path length, were performed using Gephi v.0.9.2 [61].

The Zi-score and Pi-score cut-offs, based on methodologies used for metabolic networks, were applied to classify according to their topological roles within the network [62,63]. Network hubs, module hubs, and connectors were identified as key topological features, which are considered to play important roles in the stability and resistance of microbial communities [64]. The ZOTUs corresponding to these key nodes were defined as keystone species.

### 2.6. Phylogenetic Analysis

To determine the phylogenetic affiliations of the recovered haptophyte ZOTUs and explore potential novel diversity, the 1433 environmental ZOTUs were combined with 971 haptophyte reference sequences provided by Edvardsen et al. [40]. The cryptophyte *Chroomonas mesostigmatica* (GenBank accession no. AF508268) was designated as the outgroup. Multiple sequence alignment of the combined dataset was performed using MAFFT v7.520 [65], and ambiguously aligned regions were subsequently trimmed using the ‘gappyout’ algorithm in trimAl v1.4.rev15 [66]. A maximum likelihood (ML) phylogenetic tree was then constructed using IQ-TREE v2.2.6 [67]. The best-fit nucleotide substitution model was automatically evaluated and selected by the integrated ModelFinder [68], and node support was assessed via 1000 bootstrap replicates. The final phylogenetic tree was visualized and annotated using the interactive Tree Of Life (iTOL, version 7.5.1, https://itol.embl.de, accessed on 30 January 2025) [69].

## 3. Results

### 3.1. Taxonomic Composition and Size Distribution of Haptophyte Communities

After quality filtering, 2,832,327 reads remained. Following the removal of chimeras, non-Haptophyta-affiliated ZOTUs, and singletons, 2,429,313 reads remained, ranging from 40,734 to 112,389 reads per sample. These reads were clustered into 1453 ZOTUs, with a range of 586 to 870 ZOTUs per sample. Following rarefaction to the lowest read count across all samples, 1433 ZOTUs belonging to 33 genera, 14 orders, and 6 classes were retained for downstream analysis. The pico- and nano-sized datasets contained 1350 and 1224 ZOTUs, respectively, sharing 79.7% of their ZOTUs. Rarefaction curves indicated saturation, suggesting that sequencing effort captured most of the local species diversity (Figure S1).

**Figure 2:**
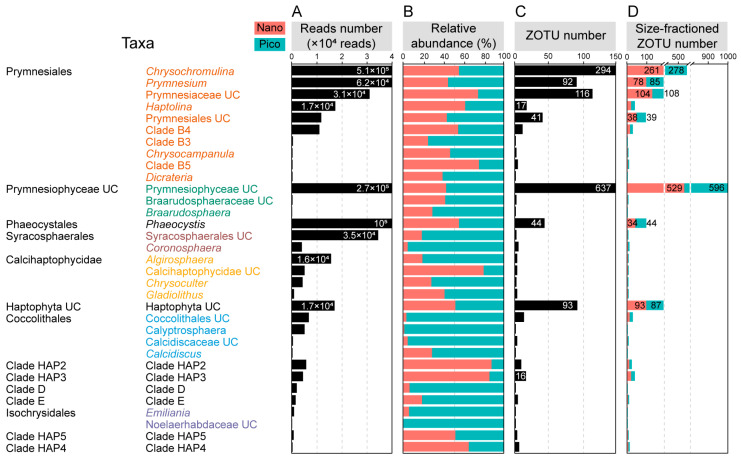
Taxonomic distribution of haptophytes. (**A**) Total sequence abundance across various haptophyte assemblages. (**B**) Relative sequence abundance of haptophyte assemblages between the nano- and pico-subcommunities. (**C**) Total number of ZOTUs assigned to different haptophyte assemblages. (**D**) Comparison of the number of ZOTUs between the nano- and pico-subcommunities. Taxa belonging to the same higher-level taxonomic group are denoted by the same color.

Of the 1433 ZOTUs, 45% were affiliated with Prymnesiophyceae_UC and 40% with Prymnesiales (Figure 2C). The pico-sized fraction contained a higher number of ZOTUs than the nano-sized fraction (Figure 2D). The genera *Chrysochromulina* and *Phaeocystis* exhibited high relative abundances but low ZOTU numbers, whereas Prymnesiophyceae_UC and Haptophyta_UC displayed the opposite pattern (Figure 2D). Sequence abundance varied considerably across taxa, ranging from 2 reads (Noelaerhabdaceae_UC) to 5.1 × 10^5^ reads (*Chrysochromulina*) (Figure 2A). The order Prymnesiales was the most abundant, comprising approximately 63% and 51% of the pico- and nano-subcommunities, respectively (Figure 2B and Figure S2B). Within Prymnesiales, *Chrysochromulina* (ca. 40% in pico- and 50% in nano-subcommunity) and *Prymnesium* (ca. 6% in pico- and 5% in nano-subcommunity) were well-represented and detected across all samples (Figure 2B). Following *Chrysochromulina*, Prymnesiopyceae_UC was the second most abundant group, exhibiting higher prevalence in the pico-subcommunity compared to the nano-subcommunity. *Phaeocystis*, another abundant genus, was almost equally represented in both fractions. The environmental clades generally displayed low sequence abundances and ZOTU numbers. However, some clades showed distinct size-specific distribution patterns. For example, Clade D, Clade E, and Clade B3 were more abundant in the pico-subcommunity, whereas Clade HAP2, Clade HAP3, and Clade B5 were more prevalent in the nano-subcommunity (Figure 2B).

**Figure 3:**
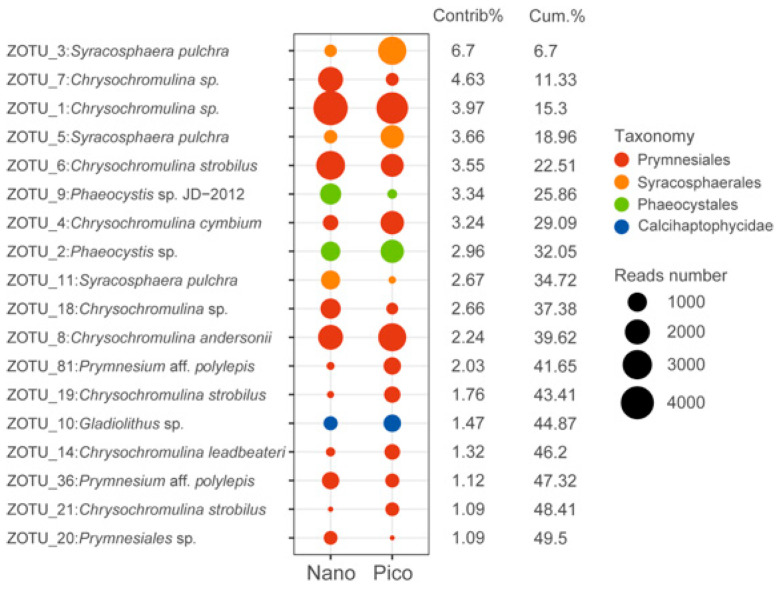
Taxonomic identities (by blasting against the NCBI database to obtain their first hit with a species name) of the 18 ZOTUs that contributed most to community dissimilarities between nano- and pico-haptophyte subcommunities with their relative contributions to each subcommunity. The diameters of the circles are proportional to the abundances of the ZOTUs in the nano- and pico-haptophyte subcommunities.

Based on SIMPER analysis, 18 ZOTUs contributed to approximately 50% of the divergence between the pico- and nano-subcommunities, with *Chrysochromulina* and *Syracosphaera* emerging as the primary taxa driving this dissimilarity (Figure 3).

### 3.2. Diversity Patterns and Community Differentiation Across Size Fractions

**Figure 4:**
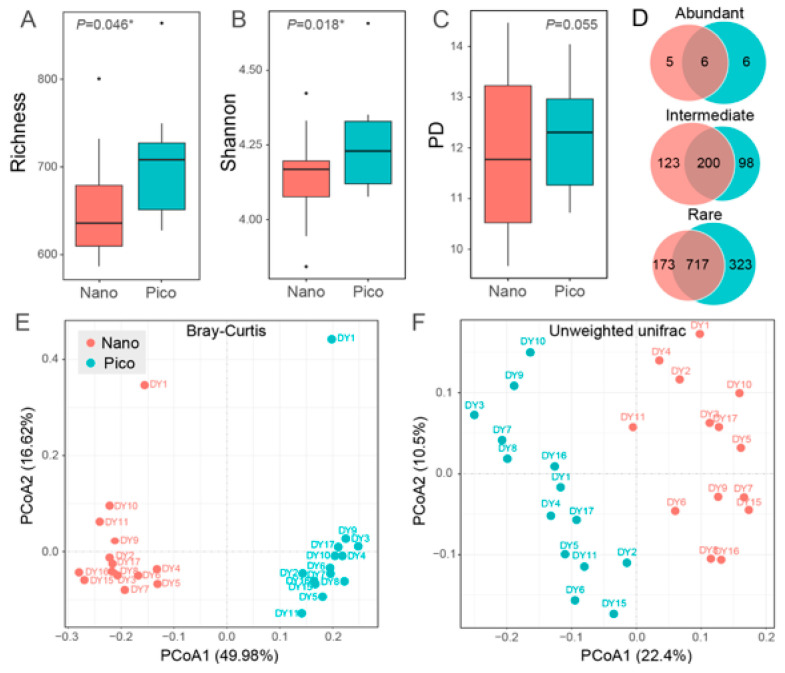
(**A**–**C**) Comparison of alpha diversity indices (ZOTU richness, Shannon, and PD) between the pico- and nano-subcommunities. Asterisk indicates statistically significant differences (* *p* < 0.05). Dots represent outliers. (**D**) Venn diagram showing the shared ZOTUs between the pico- and nano-subcommunities across the three ZOTU categories (AT, IT, and RT). (**E**,**F**) Principal coordinates analysis (PCoA) plots based on Bray–Curtis dissimilarities and unweighted UniFrac distance.

The pico-sized subcommunity exhibited significantly higher ZOTU richness and Shannon indices than the nano-sized subcommunity (*p* < 0.05, Wilcoxon test). A similar trend was observed for the PD index, although the difference was not statistically significant (*p* > 0.05, Wilcoxon test) (Figure 4A–C). No clear trends were observed for any of the three indices along the west-to-east transect for either size fraction (Figure S3C).

Principal coordinate analysis (PCoA) based on Bray–Curtis dissimilarities revealed a clear separation of the communities into two distinct groups corresponding to the nano- and pico-subcommunities (Figure 4E). Analysis based on unweighted UniFrac distances further supported the size-specific differentiation of the haptophyte communities (Figure 4F). Notably, in the PCoA plot based on Bray–Curtis dissimilarities, samples from station DY1 exhibited distinct clustering for both size fractions. Analysis of community composition along the transect showed that most dominant groups, including *Chrysochromulina*, Prymnesiophyceae_UC, *Phaeocystis*, and Haptophyta_UC, were evenly distributed. Compared to the other sites, DY1 exhibited relatively low abundances of *Prymnesium*, Clade_HAP2, *Algirophaera*, and *Chrysoculter*, alongside high abundances of Clade B4 and Calyptrosphera (Figure S3).

The majority of recovered ZOTUs were classified as RT (1040 and 890 in the pico- and nano-subcommunities, respectively), followed by IT (298 and 323, respectively). Only a small fraction was classified as AT (12 and 11, respectively) (Figure 4D, Table 1). BLASTn analysis (v.2.17) of the abundant ZOTUs revealed that most of the shared ZOTUs belonged to the genus *Chrysochromulina* (Table S1).

### 3.3. Environmental Factors Associated with Haptophyte Communities

**Figure 5:**
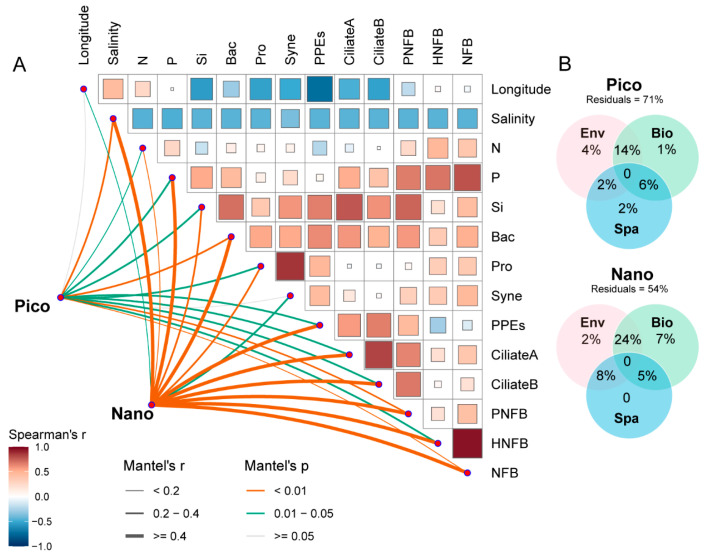
(**A**) Spearman pairwise comparisons of environmental variables and Mantel tests between the environmental variables and the pico- and nano-haptophyte subcommunities. (**B**) Variation partitioning analysis (VPA) showing the community variation explained by environmental abiotic (Env), biological (Bio), and spatial (Spa) factors. Residuals refer to the unexplained community variation. N, nitrate + nitrite nitrogen; P, phosphate phosphorus; Si, silicate; Bac, bacteria; Pro, *Prochlorococcus*; Syne, *Synechococcus*; PPEs, photosynthetic picoeukaryotes; CiliateA/B, abundance/biomass of ciliates; PNFB, biomass of pigmented nanoflagellates; HNFB, biomass of heterotrophic nanoflagellates; NFB, biomass of nanoflagellates. The block sizes in the heatmap are proportional to the absolute values of the correlation coefficients.

Community similarity within both the pico- and nano-subcommunities decreased significantly with increasing geographic distance (*p* < 0.01) (Figure S4). Salinity, bacterial abundance, pigmented nanoflagellate biomass (PNFB), and total nanoflagellate biomass (NFB) exhibited significant correlations with both the pico- and nano-subcommunities (*p* < 0.01), with stronger correlations observed in the nano-subcommunity (*r* > 0.4) (Figure 5A). Additionally, photosynthetic picoeukaryotes(PPEs), ciliate abundance (CiliateA), ciliate biomass (CiliateB), and heterotrophic nanoflagellates biomass (HNFB) showed significant and strong correlations (*r* > 0.4, *p* < 0.01) with the nano-subcommunity (Table S2). Variance partitioning analysis (VPA) showed that environmental (abiotic), biotic (food availability and predators), and spatial (geographic distance) factors collectively explained 29% and 46% of the community structure variations in the pico- and nano-subcommunities, respectively (Figure 5B).

### 3.4. Community Assembly Processes

**Figure 6:**
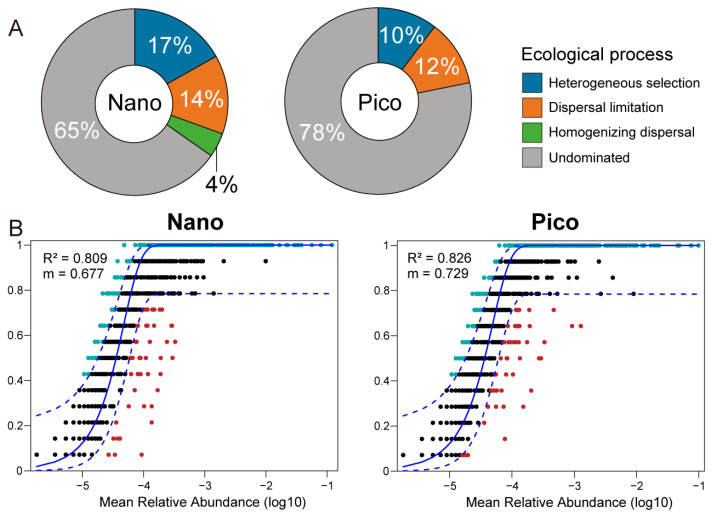
(**A**) Assembly of the nano- and pico-haptophyte subcommunities based on null model (NM) analysis. (**B**) Fitting of Sloan’s neutral community model (NCM) for the nano- and pico-haptophyte subcommunities. The solid blue lines indicate the best fit to the NCM, and the dashed blue lines represent the 95% confidence intervals around the model prediction. Different colored dots represent individual ZOTUs that occur more frequently (green dots), less frequently (red dots), or within (black dots) the neutral prediction.

Null model (NM) analysis revealed that stochastic processes, including dispersal limitation, homogeneous dispersal, and undominated processes, predominantly governed the assembly of both size fractions. Undominated processes, specifically, accounted for 78% and 65% of the community assembly in the pico- and nano-subcommunities, respectively (Figure 6A). In contrast, deterministic factors (i.e., heterogeneous selection) played a more significant role in the assembly of the nano-subcommunity compared to the pico-subcommunity (17% vs. 10%). The neutral community model (NCM) further supported these findings, explaining 82.6% and 80.9% of the community variation within the pico- and nano-subcommunities, respectively (Figure 6B). Furthermore, the pico-subcommunities exhibited a higher migration rate (*m* = 0.729) than the nano-subcommunity (*m* = 0.677), indicating a greater capacity for species dispersal.

### 3.5. Co-Occurrence Networks of the Nano- and Pico-Subcommunities

**Figure 7:**
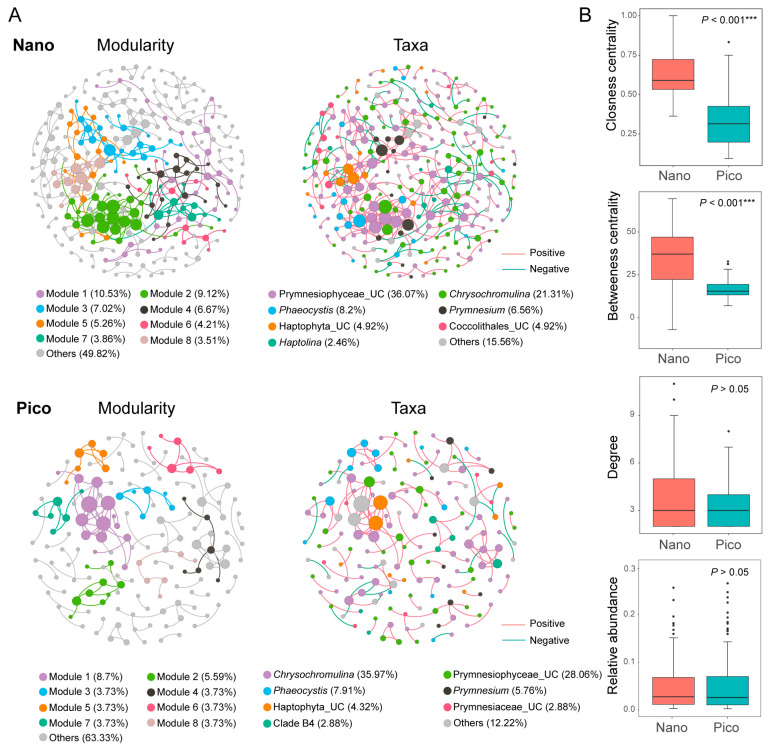
(**A**) Co-occurrence networks of nano- and pico-subcommunities. Nodes are colored according to modularity (**left**) and taxonomic identities (**right**). Node size is proportional to the degree of the ZOTU. The colors of the edges (lines) in the modularity networks (**left**) correspond to their respective modules, while the edge colors in the taxonomic networks (**right**) indicate positive or negative correlations. (**B**) Comparison of node-level topological features between nano- and pico-haptophyte subcommunities. Asterisks indicate statistically significant differences (*** *p* < 0.001). Dots represent outliers.

Co-occurrence network analysis revealed distinct structural and topological properties between the nano- and pico-subcommunities. The nano-subcommunity network comprised 285 nodes connected by 345 edges, whereas the pico-subcommunity network consisted of 161 nodes connected by 140 edges (Table S3). In the nano-subcommunity network, Prymnesiophyceae_UC was the dominant group (36.07%), followed by *Chrysochromulina* (21.31%), *Phaeocystis* (8.2%), and *Prymnesium* (6.56%). In contrast, *Chrysochromulina* (35.97%) surpassed Prymnesiophyceae_UC (28.06%) as the top contributor in the pico-subcommunity network, followed by *Phaeocystis* (7.91%) and *Prymnesium* (5.76%) (Figure 7A).

Topological analysis indicated that the pico-subcommunity network exhibited a higher proportion of positive links (85%) compared to the nano-subcommunity network (75.65%) (Table S3). The nano-subcommunity network demonstrated significantly higher betweenness centrality (*p* < 0.01, Wilcoxon test) and greater modularity. Conversely, the pico-subcommunity network was characterized by lower closeness centrality (*p* < 0.01, Wilcoxon test), a smaller network diameter, and a shorter average path length (Table S3, Figure 7B).

**Figure 8:**
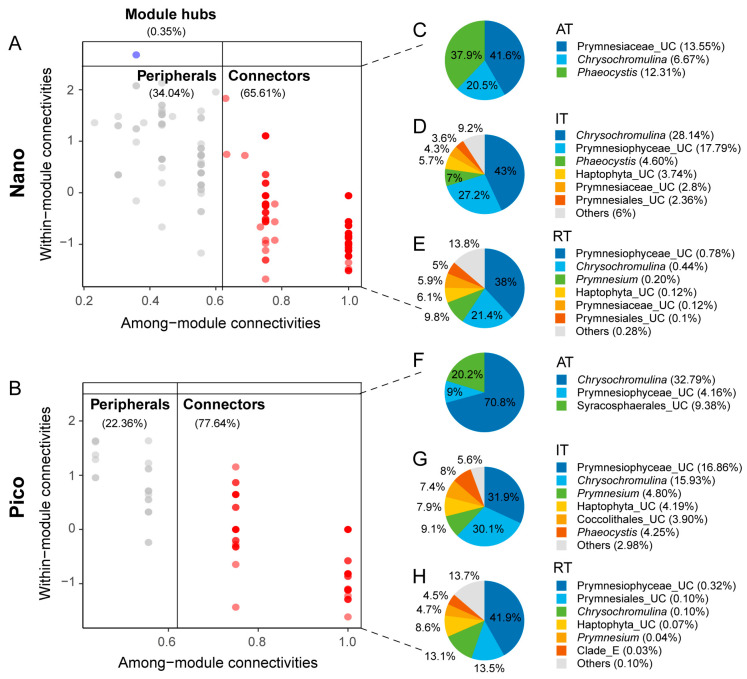
(**A**,**B**) Node classification within the nano- and pico-haptophyte subcommunity networks based on their topological roles (Zi-Pi plots) to identify putative keystone taxa. Different colors denote distinct topological roles (red for connectors and blue for module hubs). The varying shades of data points result from the overlapping of semi-transparent points, reflecting data density. (**C**–**H**) Taxonomic composition and relative abundance of the connectors in the nano- (**C**–**E**) and pico-sized (**F**–**H**) networks, categorized into AT, IT, and RT. The relative abundance of each taxon within the overall nano- or pico-sized subcommunity is indicated in parentheses following its name.

Based on the analysis of Zi and Pi values, 125 and 188 keystone species were identified in the pico- and nano-subcommunity networks, respectively (Figure S5A). In the nano-subcommunity network, 116 keystone species belonged to IT, representing 16.27% of the total nano-subcommunity sequence abundance. In contrast, 80 keystone species in the pico-subcommunity were classified as IT, accounting for 21.34% of the total pico-subcommunity sequence abundance (Figure S5). Most of these keystone species in both networks functioned as connectors, with module hubs exclusively observed in the nano-subcommunity network (specifically ZOTUs_105 and Prymnesiophyceae_UC) (Figure 8A,B). In both networks, *Chrysochromulina* and Prymnesiophyceae_UC emerged as the most important keystone taxa. These taxa accounted for 35.25% and 32.12% of the total nano-subcommunity sequences (Figure 8C–E), and 48.82% and 21.34% of the total pico-subcommunity sequences (Figure 8F–H), respectively.

Additionally, we simulated a network attack scenario to evaluate the contribution of keystone species from the AT, IT, and RT to the stability of the pico- and nano-subcommunity networks. Following the simulated removal of IT keystone species, both size-fractionated networks lost connectedness more rapidly than when RT keystone species were removed (Figure S6).

### 3.6. Phylogenetic Diversity of Haptophyta-Affiliated Sequences

**Figure 9:**
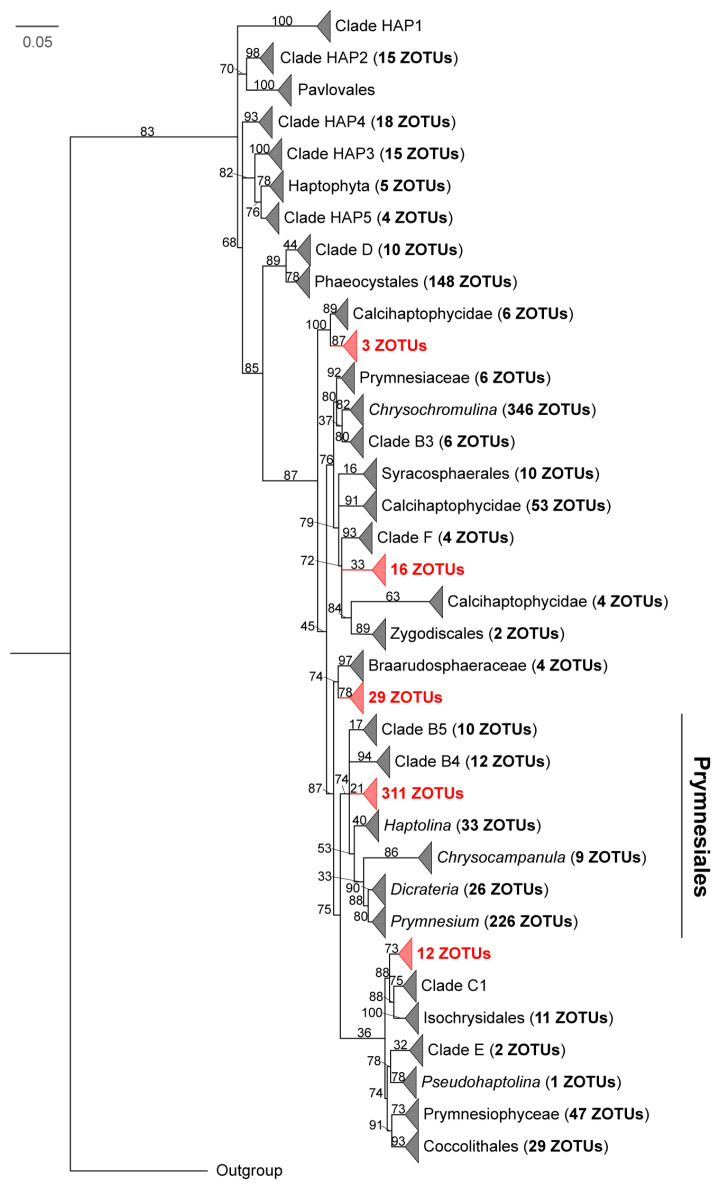
Maximum likelihood (ML) phylogenetic tree of haptophytes based on 18S rRNA gene sequences. The analysis included 1433 environmental ZOTUs recovered from the wPO in this study alongside 971 reference sequences from Edvardsen et al. [40]. Potentially novel lineages comprised exclusively of uncultured ZOTUs from this study are highlighted in red. Triangles represent collapsed clades, with the number in parentheses indicating the total ZOTUs within that lineage. *Chroomonas mesostigmatica* (AF508268) serves as the outgroup. Node support values are derived from 1000 bootstrap replicates, and the scale bar indicates the number of substitutions per site.

Phylogenetic analyses showed that most ZOTUs (74%) clustered with known reference and environmental clusters, though multiple distinct lineages consisting exclusively of environmental ZOTUs from this study were identified (Figure 9). Among the well-supported lineages, a monophyletic group of 3 ZOTUs branched near the base of the subclass Calcihaptophycidae to form a fully supported sister group. Furthermore, an independent clade of 29 ZOTUs formed a moderately supported sister group (74%) to the family Braarudosphaeraceae. At the base of the Isochrysidales lineage, which includes Clade C1, a clade of 12 ZOTUs clustered with these taxa, supported by a high bootstrap value (88%). However, the phylogenetic placement of certain clusters exhibited statistical uncertainty. For example, a cluster of 16 ZOTUs formed a paraphyletic relationship with Clade F and Calcihaptophycidae, leaving its phylogenetic relationship unresolved. Additionally, a large cluster containing 311 ZOTUs was found within the Prymnesiales lineage. While this group branched parallel to lineages comprising known taxa (e.g., Clade B4, Clade B5, and *Prymnesium*), its monophyly lacked statistical support (21%).

## 4. Discussion

### 4.1. Divergences Between Haptophyte Taxa and Size-Fractionated Communities

Haptophytes are widely distributed in global oceans, contributing 20–50% of the total biomass of pigment-containing phytoplankton [22]. Nano-sized haptophytes constitute 57–73% of all known haptophytes and have been extensively examined in previous morphological studies [8,27,38,70]. The diversity and abundance of pico-sized haptophytes have likely been underestimated due to their diminutive size, fragility, and difficulties in microscopic identification. While high-throughput sequencing of the 18S rRNA gene has uncovered substantial haptophyte diversity, DNA-based data do not accurately represent metabolic activity, whereas RNA is more directly associated with cellular functionality [71]. In this study, RNA-based sequencing of haptophyte-specific amplicons was used to investigate the active haptophyte community in the wPO.

The dominance of *Chrysochromulina* and *Prymnesium* in the haptophyte community observed in this study corresponds with findings in the northern South China Sea [72], the South China Sea [27], the Mediterranean [73], the Arctic Ocean [33], the Atlantic Ocean [29,30], and the central Pacific Ocean [74]. The prevalence and dominance of *Prymnesium* can be attributed to their eurythermal and euryhaline characteristics, allowing them to tolerate temperatures from 5 °C to 35 °C and salinities ranging from 0.5% to 35% [75]. The potential mixotrophic capabilities of both genera, which enable phagotrophy to enhance carbon acquisition, confer a competitive edge in oligotrophic, high-light surface waters [7,76,77,78].

In the present study, the pico- and nano-subcommunities were clearly separated (Figure 4E,F), which is consistent with previous studies exploring the differences between size fractionated protistan communities, including haptophytes [30,34,53,79]. SIMPER analysis showed that the differences between the pico- and nano-haptophyte subcommunities were driven largely by the distribution patterns of *Chrysochromulina* and *Syracosphaera* (Figure 3). *Chrysochromulina* was more abundant in the nano-sized fraction, while *Syracosphaera* (order Syracosphaerales) primarily consisted of pico-sized species. The calcification of *Syracosphaera* may increase cell density and accelerate sinking rates [12], whereas the smaller pico-sized cells likely enhance suspension capabilities, adapting to resource constraints in oligotrophic waters. In contrast, *Chrysochromulina*, as a non-calcifying group, may allocate more resources to mixotrophic strategies [8]. The dominance of nano-sized haptophytes in bacterivory may be attributed to their larger cell size, which facilitates the ingestion of particulate organic matter [70].

Approximately 79.7% of ZOTUs were shared between pico- and nano-subcommunities, which is similar to the result of a seasonal study of haptophytes in the coastal waters of Norway (ca. 60%) that used both size fractionated samples and RNA-based sequencing [30]. The high number of shared ZOTUs between the two fractions may be attributed to the filtration process. For instance, nano-sized cells may pass through the 3-µm filter, while some pico-sized cells may be retained on it. It is plausible that large cells squeeze through small pores more readily than small cells are retained on larger-pore filters. This dynamic could artificially inflate the diversity estimates observed in the pico-sized fraction compared to the nano-sized fraction (Figure 4A–C). Similar variations have been documented elsewhere. For example, Xu et al. examined size fractionated microbial eukaryote communities along a transect from the East Sea of Korea to the central Arctic Ocean. They found higher diversity in the pico-sized fraction at most stations outside the Arctic Ocean, whereas within the Arctic circle, the nano-sized fraction generally exhibited higher diversity estimates [34]. Additionally, Bittner et al. [79] compared the alpha diversity of pico- and nano-sized haptophytes in the subsurface water (1 m) and the deep chlorophyll maximum (DCM) layer. They found comparable Shannon estimates between the two size fractions in the subsurface water, but higher Shannon estimates in the nano-sized communities within the DCM layer. Ultimately, the high proportion of shared ZOTUs may be influenced by biological life cycles and filtration artifacts, as inconsistent filtration pressures or membrane clogging can cause cell lysis, leading to larger cellular contents being retained on smaller-pore filters [71]. Therefore, the results of alpha diversity comparisons between size fractions must be interpreted with caution.

The haptophyte communities in our study primarily consisted of rare taxa, which accounted 78% of the shared ZOTUs. This finding aligns with the “rare biosphere” hypothesis [60], which posits that low-abundance microbial taxa function as a seed bank and may become dominant under specific environmental conditions [60,80]. Previous studies have demonstrated that the diversity structure of haptophytes supports this concept [30,79].

### 4.2. Environmental Drivers of Size Fractionated Haptophyte Communities

The different responses of pico- and nano-sized haptophyte communities to environmental and biological factors underscore distinct ecological strategies between the size fractions. The stronger correlations observed between the nano-subcommunity and variables such as salinity, nutrient concentrations, bacterial abundance, PNFB, and NFB likely reflect the increased sensitivity of larger phytoplankton to resource competition and predator–prey interactions. This also suggests a stronger environmental filtering effect on this size class relative to the pico-sized subcommunity [81]. Pico-sized haptophytes, owing to their higher surface area to volume ratio, exhibit faster nutrient uptake rates [41]. Their significant correlations with PPEs and ciliate biomass further imply trophic connections: other PPEs may compete for light and inorganic nutrients, while ciliates and HNFs likely exert top-down control via selective grazing on smaller prey. These findings align with the variance partitioning analysis (VPA), which indicated that the structural disparity between the two size fractions is driven by a combination of environmental (e.g., physical factors and nutrients) and biological (e.g., food availability and grazing pressure) factors. However, the substantial proportion of unexplained variance in the VPA likely indicates the influence of unmeasured environmental variables and complex species interactions [82]. In marine ecosystems, protistan grazing and viral lysis are the primary drivers of phytoplankton mortality [83]. Although this study highlights nanoflagellate grazing as a key top-down control, viral infection was not measured and likely accounts for a portion of the unexplained variance in our VPA results. Accounting for this is crucial, as viral infection in marine phytoplankton is highly host-specific [84], and targeted viral lysis can significantly alter the haptophyte community dynamics, drive bloom termination, and influence local carbon cycling [83,85,86]. Therefore, to fully disentangle the top-down mechanisms controlling size-fractionated haptophyte communities, future environmental surveys must incorporate viral abundance or genomic data to better elucidate these intricate biological interactions.

The present study found that stochastic processes, including undominated (drift), dispersal limitation, and homogenizing dispersal, were the primary forces governing the assembly of both the pico- and nano-sized haptophyte communities (Figure 6A). A recent study examining the ecological processes shaping the latitudinal community structure of haptophytes across the Pacific Ocean found that at the basin scale, heterogeneous selection dominated assembly processes [37]. That study also observed that the contribution of heterogeneous selection decreased from subarctic to tropic regions [37]. The discrepancies between the findings of Xu et al. [37] and the present study may be attributed to the type of nucleic acid analyzed (environmental DNA based vs. the environmental RNA based sequencing used here), as these two approaches have been shown to reveal significantly different community structures for both protist and prokaryotes [87,88,89,90]. Furthermore, the differences may stem from the specific size factions analyzed (pico- and nano-communities vs. the total community inferred in by Xu et al.) [37]. Additionally, the relative contribution of ecological processes depends heavily on the spatial scales of the study, which likely contributed to the contrasting results [37,91].

Our null model (NM) analysis demonstrated that deterministic processes, particularly heterogeneous selection, exerted a significantly stronger influence on the nano-subcommunity (17%) than on the pico-subcommunity (10%) (Figure 6). This aligns with the concept that deterministic assembly is driven by both biotic and abiotic factors [57]. The integration of NM and NCM provided further insights into the relative contributions of stochastic and deterministic processes. The higher *R*^2^ value of the NCM for the pico-sized subcommunity, combined with a lower proportion of dispersal limitation (11% vs. 14% in the nano-sized subcommunity), supports the “size-plasticity hypothesis”. This hypothesis posits that smaller organisms, owing to their metabolic plasticity, undergo less environmental filtering than larger organisms [92]. The increased dispersal limitations observed in the nano-sized fraction are consistent with studies showing that dispersal constraints correlate positively with organism size [93]. Collectively, our findings suggest that both stochastic and deterministic processes influence the assembly of both size fractions. However, the nano-sized subcommunity is more strongly influenced by stochastic processes than the pico-sized fraction.

Co-occurrence network analysis revealed distinct topological features between pico- and nano-sized subcommunities. The nano-sized network exhibited stronger interconnections and greater modularity, suggesting a more robust architecture with an enhanced buffering capacity against environmental perturbations [94,95]. This structural robustness may arise from the broader niche breadth of nano-sized haptophytes, which generally possess versatile metabolic strategies to exploit fluctuating resources. Conversely, the pico-sized network displayed a higher ratio of positive interactions but a more diffuse overall structure, potentially reflecting increased sensitivity to environmental variability and a stronger reliance on keystone taxa for stability maintenance.

The pivotal role of keystone species in community richness and functionality has been previously highlighted [96,97]. In the present study, keystone species consisted primarily of connectors, and the differences in keystone species abundance and ZOTU numbers between the two size fractions were largely driven by AT and IT. These taxa likely function as bridges between and within network modules, contributing significantly to the observed structural differences between the pico- and nano-sized networks. Members of *Chrysochromulina* and Prymnesiophyceae_UC were identified as keystone species in both size fractions (Figure 8), playing key roles in connecting modules. The majority of identified keystone species were classified as IT, supporting the view that IT play a crucial role in maintaining the stability of native biological communities [98,99]. Specifically, 116 of the 188 keystone species in the nano-subcommunity network and 80 of the 125 keystone species in the pico-subcommunity network were classified as IT. Furthermore, the simulated network attack showed that both networks lost connectedness more rapidly following the removal of IT compared to the removal of RT. This indicates that IT play a more crucial role in maintaining network stability than RT, reinforcing their essential contribution to community stability and functionality [99]. Finally, although the overall diversity of the pico-sized subcommunity surpassed that of the nano-sized fraction, it contained fewer keystone species. This discrepancy suggests a higher degree of functional redundancy within the pico-sized subcommunity.

### 4.3. Potential New Haptophyte Clades

The application of culture-independent methods, such as high throughput sequencing of SSU and LSU rRNA marker genes, has significantly accelerated the discovery of novel diversity within diverse protistan groups, including haptophytes [21,30,79]. In the present study, most ZOTUs recovered were assigned to known haptophyte groups (Figure 9). The identification of multiple deep-branching, uncultured lineages, such as clades basal to Calcihaptophycidae or sister to Braarudosphaeraceae, demonstrates that morphology-based surveys continue to underestimate haptophyte diversity in the wPO [40,100]. These well-supported monophyletic groups likely represent novel, uncultured taxa at or above the family level [29,71]. Conversely, the unresolved placement of certain clusters, such as the clade of 311 ZOTU within Prymnesiales, highlights the inherent limitations of the short 18S rRNA V4 region for resolving deep phylogenetic relationships [101,102]. Although these poorly supported branches represent substantial uncharacterized genetic diversity, confirming their taxonomic status will require full-length 18S rRNA gene or phylogenomic analyses [103,104]. Notably, the detection of these potentially novel ZOTUs across both the pico- and nano-sized fractions points to the phenotypic plasticity or complex life cycles characteristic of haptophytes [105,106]. Ultimately, even with some evolutionary positions remaining unresolved, our findings confirm that size-fractionated sampling is critical for accurately capturing the ecological distribution of hidden haptophyte diversity in oligotrophic oceans [107,108].

In conclusion, this study highlights the contrasting assembly mechanisms, network topologies, and extensive hidden diversity of active pico- and nano-sized haptophyte communities in the wPO. Ecologically, while environmental factors exert a stronger influence on the nano-sized fraction, this community demonstrates a more robust, tightly connected network structure. Furthermore, the disproportionate structural role of intermediate keystone taxa underscores the critical importance of low-abundance species in sustaining ecosystem stability. Phylogenetically, the discovery of multiple deep-branching, uncultured lineages across both size fractions reveals that haptophyte diversity remains significantly underestimated and suggests the prevalence of complex life cycles or phenotypic plasticity. To build upon these findings, future studies should incorporate a broader range of environmental variables, biotic interactions, full-length 18S rRNA gene analyses, as well as morphology-based characteristics to fully resolve the complex ecological drivers and evolutionary relationships governing haptophyte dynamics.

## Figures and Tables

**Table 1:** Relative sequence abundance of unique and shared ZOTUs between the nano- and pico-haptophyte subcommunities across the different ZOTU categories (AT, IT, and RT).

	Category	Nano	Pico
Unique ZOTUs	AT	6 (20.62%)	5 (17.92%)
	IT	98 (9.03%)	123 (9.91%)
	RT	323 (1.09%)	173 (0.52%)
Shared ZOTUs	AT	6 (33.72%)	6 (32.49%)
	IT	200 (33.62%)	200 (37.32%)
	RT	717 (1.94%)	717 (1.85%)

## Data Availability

The original contributions presented in this study are included in the article/Supplementary Materials. Further inquiries can be directed to the corresponding author.

## Supplementary Materials

The following supporting information can be downloaded at: https://www.mdpi.com/article/10.3390/microorganisms14040941/s1, Figure S1: Rarefaction curves of haptophyte SSU rRNA in each sample; Figure S2: Taxonomic composition of pico- and nano-haptophytes in each sample; Figure S3: Spatial distribution of the relative abundance of haptophyte communities across the longitudinal transect. Figure S4: The distance–decay pattern between microbial community similarity and geographic distance; Figure S5: Keystone species analysis of nano- and pico-sized networks; Figure S6: Network stability in response to the targeted removal of keystone species across different abundance categories (AT, IT, and RT) in nano- and pico-haptophyte subcommunities; Table S1: The abundant taxa of nano- and pico-sized unique ZOTUs, and shared ZOTUs of both fractions; Table S2: Mantel test showing the physical, biotic, and pigment effects on the haptophyte community; Table S3: Topological properties of the nano and pico co-occurrence networks of haptophyte communities.
