# Supplementary material for: Spatial Distributions of Active Pico- and Nano-Haptophytes (Eukaryota, Hacrobia) in the Tropical and Subtropical Western Pacific Ocean
Source: Microorganisms. 2026 Apr 21;14(4):941. doi: 10.3390/microorganisms14040941 (PMC13118463; doi:10.3390/microorganisms14040941)
Supplement: Supplementary file 1 [file microorganisms-14-00941-s001.zip › microorganisms-4221622-supplementary.pdf]

microorganisms

MDPI

*Supplementary Materials*

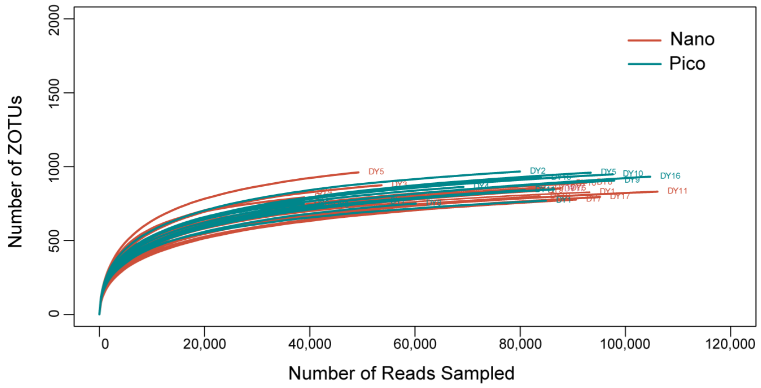


**Figure S1.** Rarefaction curves of haptophyte SSU rRNA in each sample.

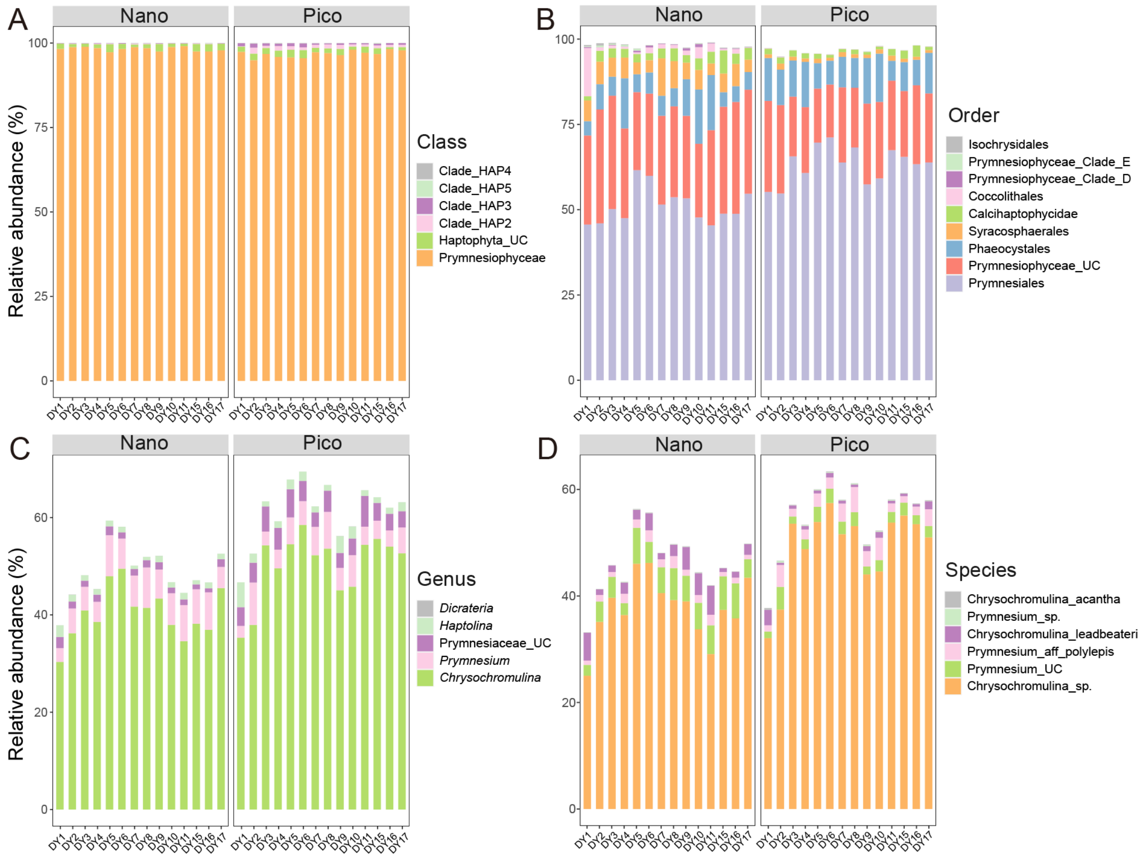


**Figure S2.** Taxonomic composition of pico- and nano-haptophytes in each sample. Proportions of sequence abundance of haptophytes on the class level (**A**), within Prymnesiophyceae (**B**), within Prymnesiales (**C**), and within *Chrysochromulina* and *Prymnesium* (**D**).

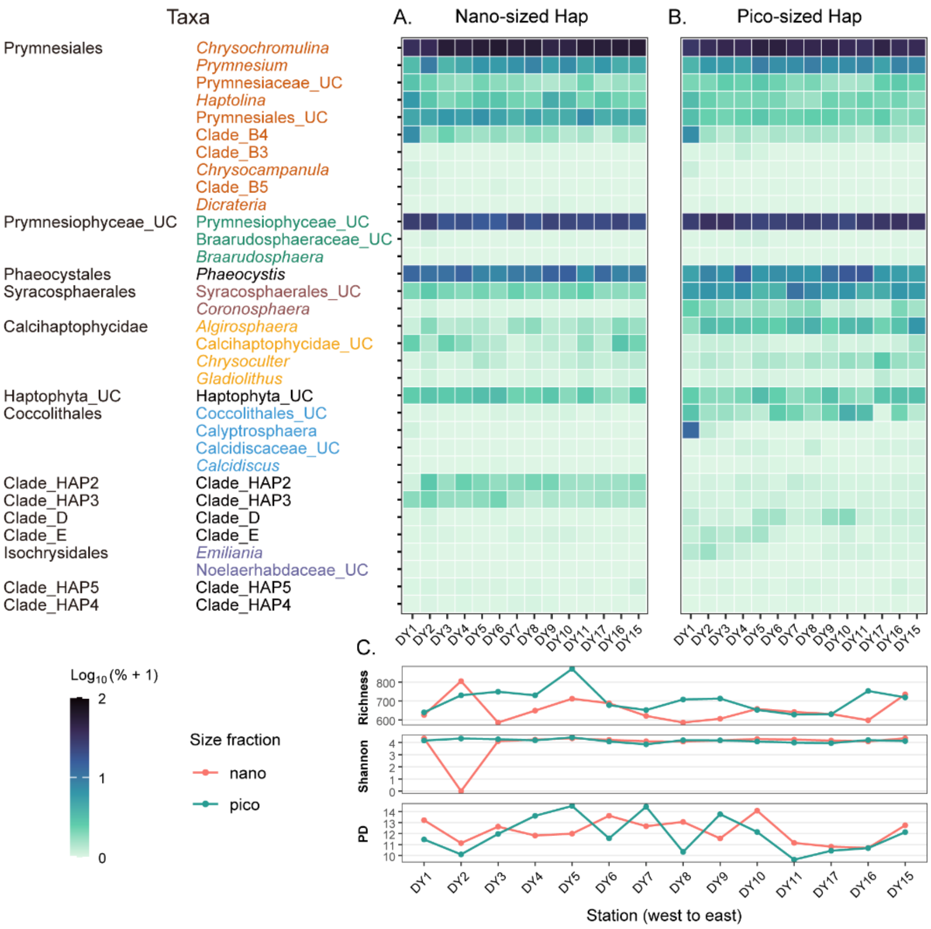


**Figure S3.** Spatial distribution of the relative abundance of haptophyte communities across the longitudinal transect. Heatmaps display the taxonomic composition of (**A**) nano- and (**B**) pico- haptophyte assemblages at the genus or clade level. (**C**) Distribution of the alpha diversity indices for both size fractions along the sampling transect. Sampling stations are ordered geographically from West to East. Color gradients represent the Log10-transformed relative abundances (Log10 (% + 1)).

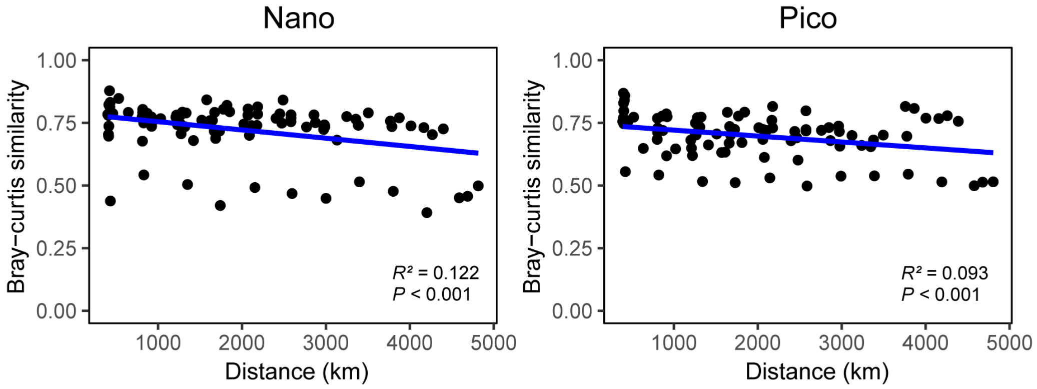


**Figure S4.** The distance-decay pattern between the community similarity of nano- (**left**) and pico-sized (**right**) haptophytes and geographic distance. Black dots represent pairwise comparisons between samples. The solid blue lines indicate the linear regression fits.

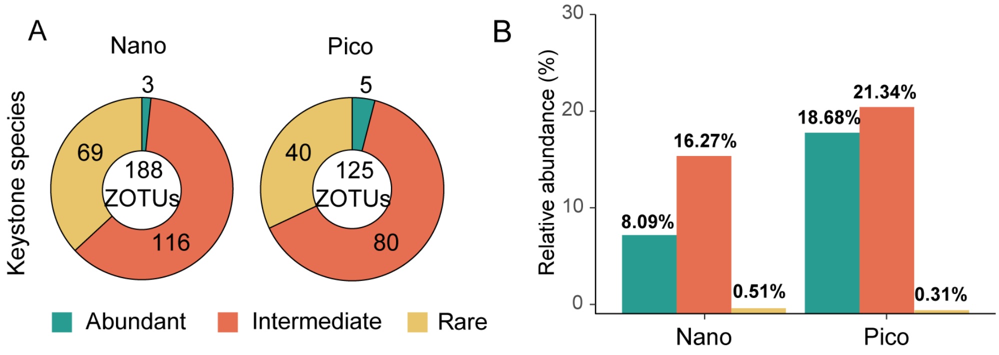


**Figure S5.** Keystone species analysis of nano- and pico-sized networks. (**A**) Total number of connectors in the nano- and pico- sized networks, along with their distribution across three ZOTUs categories. (**B**) Relative abundance of connectors in the nano- and pico-sized networks.

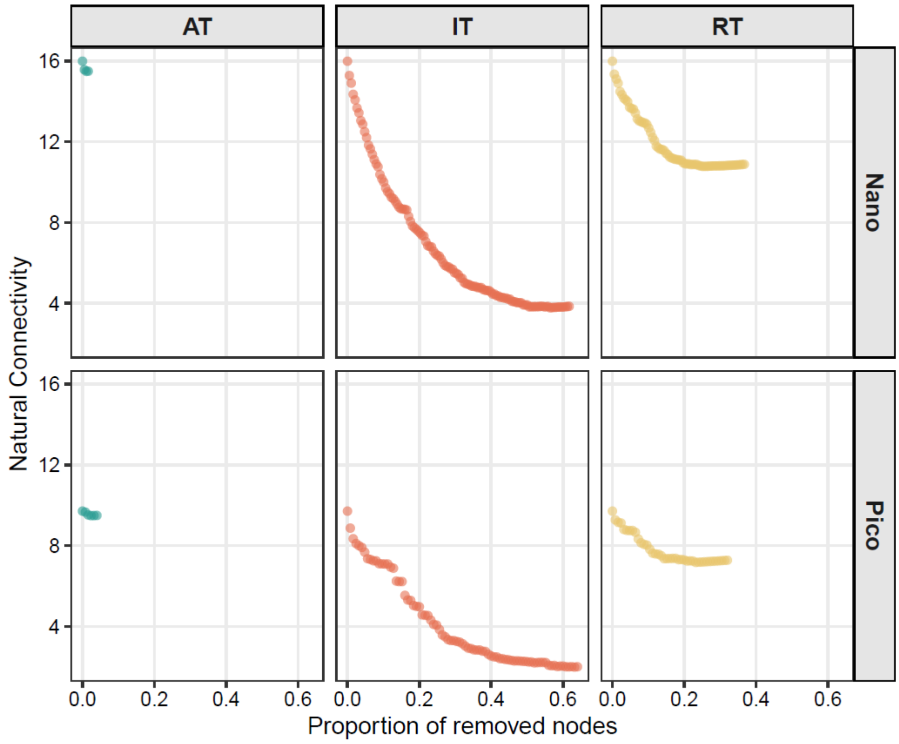


**Figure S6.** Network stability in response to the targeted removal of keystone species across different abundance categories (AT, IT, and RT) in nano- and pico-haptophyte subcommunities.

**Table S1.** The abundant taxa of nano and pico-sized unique ZOTUs, and shared ZOTUs of both fractions.

| Size Fraction | ZOTU ID | Relative Abundance | BLAST | Identity | Accession |
|---|---|---|---|---|---|
| Nano | ZOTU 3 | 7.61% | *Syracosphaera pulchra* | 100% | KF422621 |
| Nano | ZOTU 5 | 4.78% | *Syracosphaera pulchra* | 100% | KF422621 |
| Nano | ZOTU 10 | 2.29% | Haptophyta sp. | 100% | KR338487 |
| Nano | ZOTU 14 | 1.71% | Prymnesiophyceae sp. | 100% | MZ687705 |
| Nano | ZOTU 19 | 1.95% | uncultured eukaryote | 100% | KU743849 |
| Nano | ZOTU 81 | 2.29% | *Azadinium trinitatum* | 100% | LS974170 |
| Pico | ZOTU 7 | 5.63% | *Chrysochromulina* sp. | 100% | MZ611704 |
| Pico | ZOTU 9 | 3.79% | *Phaeocystis* sp. JD-2012 | 97.49% | JX660995 |
| Pico | ZOTU 11 | 2.89% | Haptophyta_UC | 100% | MG983557 |

| | | | | | |
|---|---|---|---|---|---|
| Pico | ZOTU 18 | 3.37% | *Chrysochromulina* sp. | 99.72% | MZ611704 |
| Pico | ZOTU 36 | 2.25% | *Prymnesium* sp. | 100% | MZ687721 |
| Both | ZOTU 1 | 10.97% | Haptophyta_UC | 100% | MZ687671 |
| Both | ZOTU 2 | 3.92% | *Phaeocystis*_sp. | 98.88% | JX660995 |
| Both | ZOTU 4 | 3.26% | *Chrysochromulina cymbium* | 100% | AM491018 |
| Both | ZOTU 6 | 6.26% | *Chrysochromulina strobilus* | 100% | KY980316 |
| Both | ZOTU 8 | 6.55% | *Chrysochromulina andersonii* | 99.72% | AB180202 |
| Both | ZOTU 82 | 2.15% | *Chrysochromulina andersonii* | 98.4% | AB180202 |

BLAST: the nearest BLAST hit in NCBI-nr to each unique abundant ZOTU. The relative abundance of shared ZOTUs is the mean of the relative abundance of the corresponding ZOTU in both the nano- and pico-sized samples.

**Table S2.** Mantel test showing the correlations between the environmental factors and the haptophyte community.

| **Environmental Factors** | **Nano** | | **Pico** | |
|---|---|---|---|---|
| | ***r*** | ***p*** | ***r*** | ***p*** |
| Geographical distance | 0.083 | 0.023 | 0.049 | 0.168 |
| Temperature | 0.010 | 0.496 | 0.080 | 0.677 |
| Salinity | 0.789 | 0.007 | 0.930 | 0.047 |
| N | 0.336 | 0.009 | 0.195 | 0.048 |
| P | 0.731 | 0.005 | 0.336 | 0.046 |
| Si | 0.382 | 0.002 | 0.377 | 0.030 |
| Bacteria, abundance | 0.476 | 0.006 | 0.300 | 0.009 |
| *Prochlorococcus*, abundance | 0.366 | 0.005 | 0.370 | 0.047 |
| *Synechococcus*, abundance | 0.241 | 0.036 | 0.456 | 0.128 |
| PPEs, abundance | 0.683 | 0.003 | 0.258 | 0.030 |
| Ciliate, abundance | -0.356 | 0.005 | -0.206 | 0.035 |
| Ciliate, biomass | -0.308 | 0.005 | -0.255 | 0.043 |
| PNFA | 0.005 | 0.379 | 0.138 | 0.753 |
| PNFB | 0.753 | 0.004 | 0.310 | 0.043 |
| HNFA | 0.118 | 0.224 | 0.206 | 0.143 |
| HNFB | 0.130 | 0.006 | 0.131 | 0.008 |
| NFA | 0.027 | 0.328 | 0.215 | 0.953 |
| NFB | 0.778 | 0.001 | 0.205 | 0.029 |
| Phytoplankton pigments | | | | |
| Chl *a* | -0.119 | 0.969 | -0.063 | 0.768 |
| Chl *b* | -0.099 | 0.885 | -0.047 | 0.663 |
| Chl *c3* | 0.101 | 0.874 | 0.066 | 0.756 |
| Chl *c2* | 0.104 | 0.922 | 0.051 | 0.693 |
| Peridinin | -0.057 | 0.677 | -0.021 | 0.507 |
| 19 but fucoxanthin | 0.084 | 0.806 | 0.036 | 0.567 |
| 19 hex fucoxanthin | 0.047 | 0.245 | -0.004 | 0.464 |
| Fucoxanthin | 0.070 | 0.212 | 0.028 | 0.284 |
| Neoxanthin | -0.099 | 0.893 | -0.047 | 0.640 |
| Violaxathin | 0.030 | 0.328 | 0.008 | 0.384 |
| Diadinoxanthin | -0.109 | 0.933 | -0.109 | 0.979 |
| Alloxathin | -0.052 | 0.607 | -0.089 | 0.894 |
| Zeaxanthin | -0.002 | 0.407 | -0.031 | 0.661 |
| DV.chlorophyll *a* | -0.064 | 0.691 | -0.058 | 0.751 |

**Table S3.** Topological properties of the nano- and pico-sized co-occurrence networks of haptophyte communities.

| **Index** | **Nano** | **Pico** |
|---|---|---|
| Nodes | 285 | 161 |
| Edges | 345 | 140 |
| Average degree | 2.421 | 1.739 |
| Network diameter | 17 | 6 |
| Network density | 0.009 | 0.011 |
| Modularity | 0.934 | 0.893 |
| Average clustering coefficient | 0.515 | 0.386 |
| Average path length | 5.024 | 1.864 |
| Positive | 75.65% | 85% |
| Negative | 24.35% | 15% |
